# 

**Published:** 1881-05

**Authors:** 


SEE "OUR REMOVAL TO NEW YORK," PAGE 296.

VOL. II. MAY, 1881. NO. 5.

THE

### Independent Practitioner:

A MONTHLY JOURNAL,

DEVOTED TO

***MEDICAL, SURGICAL, OBSTETRICAL, DENTAL, HYGIENICAL AND POPULAR SCIENCES.***

EDITED BY

HARVEY L. BYRD, A. M., M. D.,

AND

BASIL M. WILKERSON, D. D. S., M. D.

**"Altera poscit opem res, et conjurat amice."**

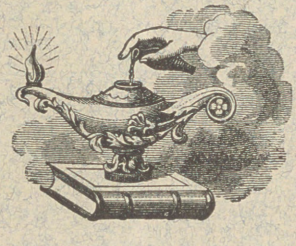

BALTIMORE;

*Published by B. M. WILKERSON, Proprietor,*

Office, 68 N. Charles Street.

WM. MILLER, Business Manager.

$3.00 Per Annum in Advance. - - Single Copies 30 Cents.

PRINTED BY THOMAS & EVANS, 9, 11, & 13 S. HOLLIDAY ST., BALTO.

